# pH induced conformational alteration in human peroxiredoxin 6 might be responsible for its resistance against lysosomal pH or high temperature

**DOI:** 10.1038/s41598-021-89093-8

**Published:** 2021-05-06

**Authors:** Rimpy Kaur Chowhan, Sunaina Hotumalani, Hamidur Rahaman, Laishram Rajendrakumar Singh

**Affiliations:** 1 Dr. B. R. Ambedkar Center for Biomedical Research, University of Delhi, Delhi, 110007 India; 2 Department of Biotechnology, Manipur University, Imphal, 795003 India

**Keywords:** Molecular conformation, Supramolecular assembly, Oxidoreductases

## Abstract

Peroxiredoxin 6 (Prdx6), the ubiquitously expressed enzyme belonging to the family of peroxidases, namely, peroxiredoxins, exhibits a unique feature of functional compartmentalization within cells. Whereas, the enzyme localized in cytosol shows glutathione peroxidase activity, its lysosomal counterpart performs calcium independent phospholipase A2 (aiPLA2) activity. Like any true moonlighting protein, these two activities of Prdx6 are mutually exclusive of each other as a function of the pH of the cellular compartments. Differential substrate preference at different pH (i.e. peroxidised phospholipids at neutral pH and reduced phospholipids at acidic pH) is considered to be the reason for this behavior. To gain insight into the pH-induced structural–functional interplay we have systematically evaluated conformational variations, thermodynamic stability of the protein and quaternary state of the conformers at both pH 7.0 and 4.0. Our findings suggest that change in pH allows alterations in native states of Prdx6 at pH 7.0 and 4.0 such that the changes make the protein resistant to thermal denaturation at low pH.

## Introduction

Peroxiredoxins (Prdx) are a group of six types of antioxidant enzymes, namely Prdx1-6, that work by reducing cellular peroxide content. This peroxidase activity is mediated by a reduced cysteine residue (also referred to as peroxidatic Cys) that donates its electron to neutralize peroxide and then recycle back into its native form with the help of reductants like thioredoxin (Prdx1-5) and glutathione (Prdx6). Exceptionally, Prdx6 is the only member of this family with additional moonlighting activity of calcium independent phospholipase A2 (aiPLA_2_), and lysophosphatidylcholine acyl transferase (LPCAT) activity. The aiPLA_2_ and LPCAT activity is possible because of the presence of lipase motif (GDSWG) that allows Prdx6 to bind with phospholipids^1,2^. Like any true moonlighting proteins, the two active sites for peroxidase (H39, C47, R132) and aiPLA_2_ (H26, S32, W33, D140) in Prdx6 are functionally independent of each other such that the deletion of one does not affect the catalytic efficiency of other^3,4^.

Mammalian Prdx6 is primarily a cytosolic protein (where peroxidase activity is maximal) and is translocated to the acidic organelles like lysosomes and lamellar bodies^1,5^ (for its aiPLA_2_ activity) with the help of 14-3-3ε chaperone^1,6,7^. This post-translational process requires direct interaction between the specific peptide sequence (amino acids 31–40) on Prdx6 and the chaperone protein, 14-3-3ε^1,6,7^. This pH specificity has been attributed to the differential substrate preference at different pH as Prdx6 at acidic pH has binding affinity for reduced phospholipids and for peroxidised phospholipids at cytosolic pH^8^. This is compatible with the biological role of Prdx6 in lipid metabolism (in lysosome) and antioxidation (in cytosol). To date much effort have been made toward understanding the pH-dependent enzymatic behaviors^1,8–10^. However, the structural alterations responsible for this pH dependent functional behaviour of Prdx6 have yet not been thoroughly explored. Therefore, structure–function relation of this enzyme is not known. In the present study to comprehend the structure–function dynamics of Prdx6, we have investigated the pH-induced conformational variations in human Prdx6 (hPrdx6). We observed that low pH brings conformational changes to switch to high order oligomer and the oligomer formation is the rationale for resistance of hPrdx6 at lysosomal pH and high temperature.

## Materials and methods

### Materials

Trizma Base, EDTA, sodium hydroxide, sodium dodecyl sulphate, glacial acetic acid, ethidium bromide, imidazole, sodium chloride, potassium chloride, glycerol, acrylamide, bis-acrylamide, ammonium persulphate, TEMED, glycine, bovine serum albumin, β-mercaptoethanol, hydrogen peroxide (H_2_O_2_), 8-anilino-1-naphthalenesulfonic acid ammonium salt (ANS), reduced glutathione (GSH), glutathione reductase, reduced β-nicotinamide adenine dinucleotide phosphate (β-NADPH), dithiothreitol (DTT), guanidinium chloride (GdmCl), luria bertani medium and luria bertani agar (BD Difco, USA), PBS tablets (Bio Basic), Ni-NTA resin (Qiagen GmbH, Germany), DNA markers (GeneDireX Inc., USA), precision dual colour protein marker (Bio-Rad), ampicillin and kanamycin (MP Biomedicals), HiYield PlusTM plasmid mini isolation kit (RBC Bioscience), bradford protein estimation kit (MP Biomedicals), EnzCheck phospholipase A2 assay kit (Invitrogen). Primary (monoclonal anti- hPrdx6, clone 3A10-2A11 antibody produced in mouse, WH0009588M1) and secondary (anti-mouse IgG (Fab Specific)-peroxidase antibody produced in goat, A3682) antibody procured from Sigma-Aldrich, *E.coli* strains: DH5α and M15[pREP4].

### Human Prdx6 overexpression and purification

The plasmid construct containing gene for hPrdx6 was purchased from Thermo Fisher Scientific. The fragment was inserted into expression vector pQE30-Xa using restriction digestion by BamHI and HindIII followed by a ligation reaction with DNA ligase. The plasmid DNA was purified from transformed *E.coli* DH5α cells and concentration determined by UV spectroscopy. The final construct and open reading frame was verified using 5′CCCGAAAAGTGCCACCTG 3′ primer for forward sequencing and 3′GGTCATTACTGGAGTCTTG5′ primer for reverse sequencing. The sequence congruence within the insertion sites was 100%. For Protein induction, *E. coli* M15[pREP4] cells transformed with pQE30-Xa/hPrdx6 construct and selected using ampicillin (100 μg/ml) and kanamycin (50 μg/ml), were treated with 0.5 mM IPTG at 37 °C. Cell extract containing hPrdx6 protein was collected after cell lysis using lysis buffer (20 mM Tris (pH 7.0), 50 mM NaCl, 10 mM Imidazole, 0.5 mg/ml lysozyme, 0.005 mg/ml RNaseA, 0.005 mg/ml DNase) and sonication (12 pulses of 30 s). The cell lysate so obtained was centrifuged and hPrdx6 was collected in the soluble supernatant fraction. For hPrdx6 protein purification, the crude protein extract was passed through the Ni-NTA agarose column pre-equilibrated with binding buffer (20 mM Tris (pH 7.0), 50 mM NaCl, 10 mM Imidazole), followed by washing and elution using wash buffer (20 mM Tris (pH 7.0), 50 mM NaCl, 60 mM Imidazole) and elution buffer (20 mM Tris (pH 7.0), 50 mM NaCl, 500 mM Imidazole), respectively. The protein-containing elution fractions were pooled and dialyzed against dialysis buffer (20 mM Tris (pH 7.0), 50 mM NaCl). The purity of the hPrdx6 was checked on SDS-PAGE (Supplementary image S1) followed by immunoblotting with respective anti-hPrdx6 antibody (as described previously^11^).

### GdmCl-induced chemical unfolding

GdmCl-induced denaturation of 0.1 g/l hPrdx6 at pH 7.0 (10 mM Tris–HCl buffer) and pH 4.0 (10 mM sodium acetate buffer) was followed by measuring changes in λ_max_ as a function of GdmCl concentration (0-5 M) at 20 °C in Cary Eclipse Fluorescence Spectrophotometer (Agilent Technologies) with an external constant temperature circulator/bath. Samples were incubated with GdmCl for 1 h before measurements. To monitor λ_max_, hPrdx6 protein was excited at 295 nm and the emission spectra were recorded from 300 to 600 nm. The fraction of the protein that is denatured at each GdmCl concentration is measured by λ_max_ using the following equation:

$${\text{f}}_{D}=\frac{{Y}_{obs}-{Y}_{n}}{{Y}_{d}-{Y}_{n}}$$

where, f_D_ is fraction denatured, Y_obs_ is the observed quantity (λ_max_), and Y_n_ and Y_d_ are the values for the native and fully denatured proteins at each of the GdmCl concentrations, respectively. f_D_ was then plotted against [GdmCl], the molar concentration of the denaturant, and chemical denaturation mid-point is measured using Origin 7.0 software.

### Heat-induced thermal unfolding

Thermal denaturation studies of hPrdx6 at different pH conditions (with buffers ranging from pH 2.0 to 10.0 as described above) were carried out in Jasco J-810 spectropolarimeter equipped with a Peltier-type temperature controller at a heating rate of 1 °C per min as per methods described in^12^. This scan rate was found to provide adequate time for equilibration. Each sample was heated from 20 to 85 °C. The change in ellipticity with increasing temperature was followed at 222 nm for 16 μM hPrdx6. Measurements were repeated three times. After denaturation, the protein sample was immediately cooled down to measure reversibility of the reaction. For the same purpose, Far-UV CD spectra for hPrdx6-before and after melting were also recorded. Each heat-induced transition curve was analysed for midpoint of denaturation (Tm) using Origin 7.0 software.

### Circular dichroism measurements

CD measurements were made in a Jasco J-810 spectropolarimeter equipped with a Peltier-type temperature controller (working range 10–90 °C) with six accumulations as per recommendations in^13^. The hPrdx6 concentration used for the CD measurements was 0.4 g/l (16 µM). Cells of 0.1 and 1.0 cm path length were used for the measurements of the far- and near-UV spectra, respectively. Necessary blanks were subtracted. The CD instrument was routinely calibrated with D-10-camphorsulfonic acid. Secondary structure estimation from the Far-UV CD spectra was calculated using Yang’s method (https://www.ncbi.nlm.nih.gov/pubmed/4366945)^11–13^. Spectra of purified hPrdx6 were recorded in different pH conditions, and the buffers used were 10 mM glycine (pH 2.0–3.0), 10 mM sodium acetate (pH 4.0–5.0), 10 mM sodium cacodylate (pH 6.0), or 10 mM Tris–HCl (pH 7.0–10.0). Each spectrum was repeated at least three times and mean curve was plotted using SigmaPlot Software 10.0.

### Tryptophan (Trp) fluorescence spectroscopy

Fluorescence spectra of the hPrdx6 (0.1 mg/ml) were measured in the presence of buffers ranging from pH 2.0 to 10.0. Buffers used were 10 mM glycine (pH 2.0–3.0), 10 mM sodium acetate (pH 4.0–5.0), 10 mM sodium cacodylate (pH 6.0), or 10 mM Tris–HCl (pH 7.0–10.0). A Cary Eclipse Fluorescence Spectrophotometer (Agilent Technologies) with both excitation and emission slits set at 10 nm in a 3 mm quartz cuvette was used. For fluorescence measurements, hPrdx6 protein was excited at 295 nm, while the emission spectra were recorded from 300 to 600 nm. Each spectrum was repeated at least three times and the mean curve was plotted using SigmaPlot Software 10.0.

### ANS binding assay

For ANS binding experiments, fluorescence emission spectra were recorded in the region of 400–600 nm and the excitation wavelength was set at 350 nm^14^. ANS concentration used was 16 times higher as that of protein concentration. Concentration of ANS was determined experimentally using *ε*, the molar absorption coefficient value of 5000/M/cm at 350 nm. All the samples were incubated for 30 min after adding ANS. Each spectrum was recorded at least three times and mean curve was plotted using SigmaPlot 10.0.

### Dynamic light scattering

Size distribution of the particles present in the protein sample was obtained using a Zetasizer Micro V/ZMV 2000 (Malvern, UK) as per methodology used in^13^. Measurements were made at a fixed angle of 90° using an incident laser beam of 689 nm. Fifteen measurements were made with an acquisition time of 30 s for each sample at sensitivity of 10%^11–13^. The data was analysed using Zetasizer software provided by the manufacturer to obtain hydrodynamic diameter aka z-average diameter (intensity weighted mean diameter derived from cumulant analysis), polydispersity index (dimensionless measure of the broadness of the size distribution calculated from cumulant analysis) and volume fraction (relative amount of different sized particles in separate peaks in a sample solution)^15^. The protein concentration used was 1.0 mg/ml.

### Size exclusion chromatography

To determine the oligomeric nature of hPrdx6 at different pH, size-exclusion chromatography (SEC) was performed using Sephadex G-100 column (GE Healthcare). The sephadex powder swelling and gel chromatographic column (2 cm diameter, 14 cm height, 44 ml bed volume) preparation was done as per manufacturer recommendations. The hPrdx6 (1 mg/ml) at pH 7.0 and pH 4.0 was subjected to SEC, equilibrated with 3 bed volumes of 10 mM Tris–HCl buffer (pH 7.0), and 10 mM sodium acetate buffer (pH 4.0), respectively. Protein elution profiles on SEC were monitored by measuring the absorbance at 280 nm with buffers at corresponding pH used as blank. To estimate void volume of the column, the elution profile of blue dextran was also monitored at 600 nm.

### Hydrogen peroxide decay assay for glutathione peroxidase activity measurement

Glutathione peroxidase activity of hPrdx6 at different pH conditions was assayed by measuring consumption of hydrogen peroxide in the presence of GSH as an electron donor^16^. The standard reaction buffer was constituted by mixing 10 mM base buffer, 2 mM NaN_3_, and 0.2 mM GSH. To test pH dependence, base buffers used were glycine (pH 2.0–3.0), sodium acetate (pH 4.0–5.0), sodium cacodylate (pH 6.0), and Tris–HCl (pH 7.0–10.0). For this end-point assay^16^, conducted at room temperature, the hPrdx6 (6 μg of protein/ml) was pre-incubated with reaction buffer with continuous stirring. Thereafter, the reaction was initiated by adding 250 μM H_2_O_2_, and the change in absorbance at 240 nm was recorded for 10 min. The results obtained were corrected for any basal non-enzymatic peroxide reduction. Enzymatic activity of hPrdx6, expressed as nmol of H_2_O_2_ reduced per min per mg of protein, was calculated using following equation:

$$Enzyme\;activity = \frac{{\Delta Abs{\text{/min}}}}{\left[ E \right]} \times 10^{3}$$

The enzyme assay was carried out at least three times.

### FRET assay for calcium independent phospholipase A_2_ activity measurement

Phospholipase A_2_ represents a family of enzymes that hydrolyze the sn-2 ester linkage of phospholipids. The assay we used to detect aiPLA_2_ activity of hPrdx6 is based on measuring distinct fluorescence resonance energy transfer (FRET) emission of the substrate prior to and after cleavage in the absence of Calcium. Samples (hPrdx6), positive controls (0–10 Units/ml of PLA_2_ from honey bee venom) and negative control (no PLA_2_) have been prepared as per manufacturer’s recommendations (EnzChek Phospholipase A2 Assay Kit, Invitrogen). For each reaction, 100 μg of purified enzyme (diluted in 10 mM base buffer) in a total volume of 50 μl is incubated with equal volume of substrate-liposome mix (prepared by mixing of 30 μl 10 mM Dioleoylphosphatidylcholine, 30 μl 10 mM Dioleoylphosphatidylglycerol, and 30 μl 1 mM PLA_2_ substrate) for 10 min at room temperature. To test pH dependence, base buffers used were glycine (pH 2.0–3.0), sodium acetate (pH 4.0–5.0), sodium cacodylate (pH 6.0), and Tris–HCl (pH 7.0–10.0). Fluorescence emission is measured at ~ 515 nm and ~ 575 nm (excitation at 460 nm) using TECAN microplate reader. Enzyme activity is quantified by comparative ratiometric analysis of samples and positive control’s fluorescence emission at 515 nm/575 nm. The enzyme assay was carried out at least three times.

## Results

### Human Prdx6 is thermodynamically more stable at low pH than at pH 7.0

**Figure 1:**
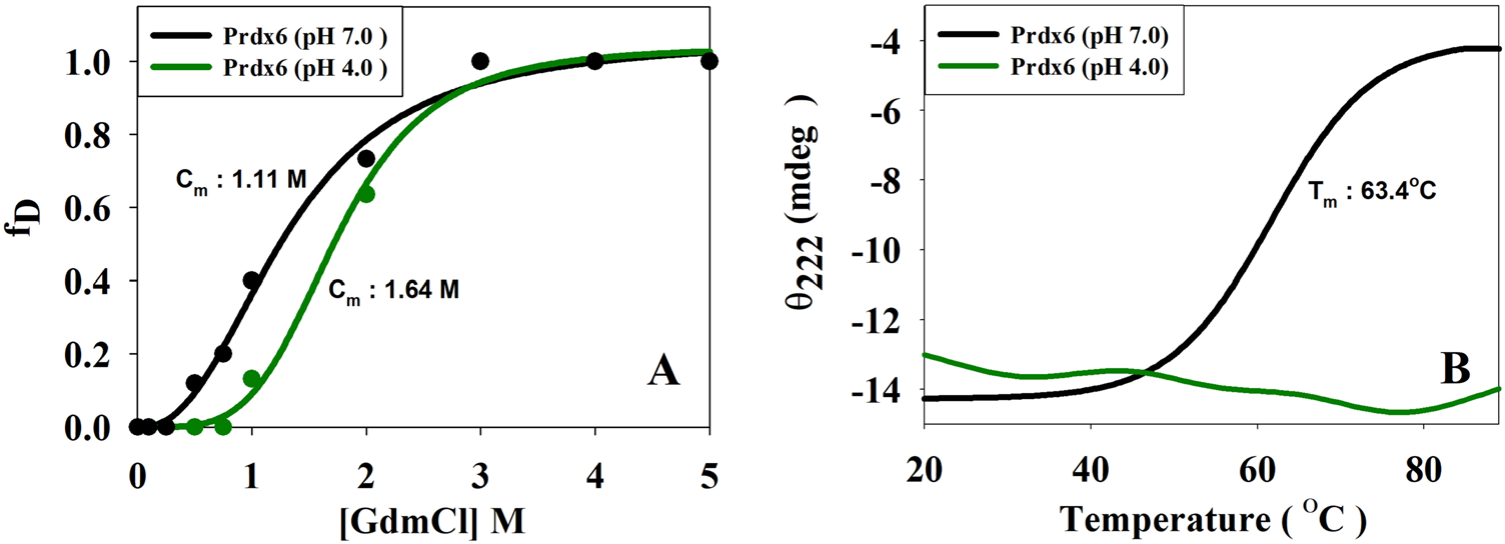
Stability of hPrdx6. The effect of cytosolic (pH 7.0) to lysosomal (pH 4.0) switching on the (**A**) thermal* and (**B**) chemical stability of hPrdx6 is determined using Far-UV CD spectroscopy. (Tm/pH7: 63.37 °C) (Cm/pH7: 1.11 M; pH4: 1.64 M).

The variations in thermodynamic stability (if any) of hPrdx6 at neutral and acidic pH were investigated by undergoing denaturation using two different modes (heat and GdmCl). Denaturation was monitored by observing changes in ellipticity at 222 nm as a function of temperature or GdmCl concentration. Figure 1A,B shows the GdmCl-induced and heat-induced denaturation profiles of hPrdx6 at pH 7.0 and 4.0. It is seen in this figure that hPrdx6 at low pH exhibit more stability as the Cm has been shifted to higher value at pH 4.0 relative to 7.0 (from 1.11 to 1.64 M). Interestingly, in the case of heat-induced denaturation at pH 4.0, there is no significant alteration in the observed θ_222_ as a function of temperature (20–85 °C) indicating that the protein has not undergone unfolding up to the measurable temperature range. There was no further change in θ_222_ upon increasing the temperature upto 95 °C (data not shown). However, at physiological pH, the measured *T*_m_ is 63.4 °C. The results indicate that hPrdx6 at pH 4.0 is resistant to thermal denaturation.

### Human Prdx6 displays altered secondary and tertiary structure at acidic pH as compared to neutral pH

**Figure 2:**
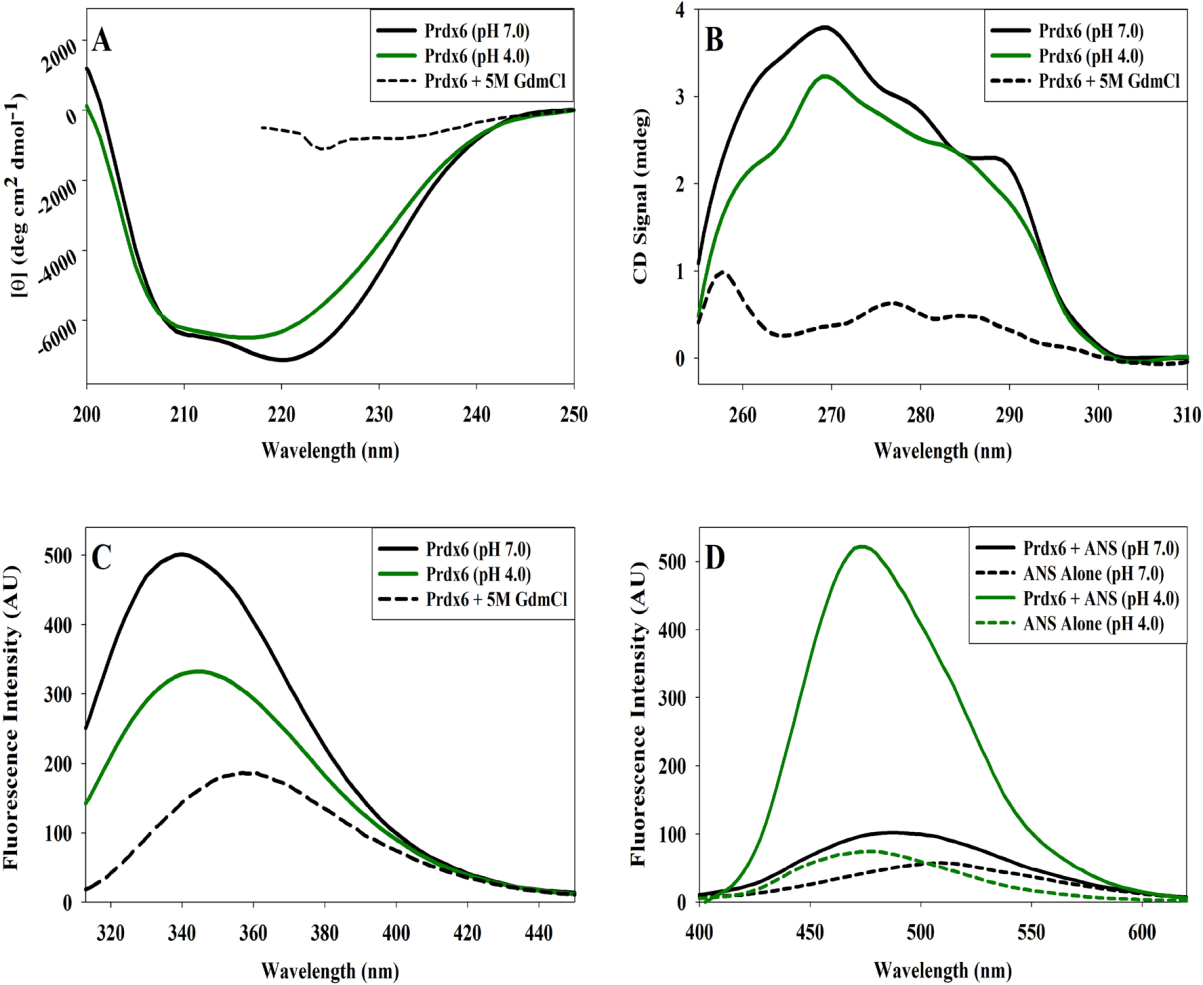
hPrdx6’s structural characterization. To determine the structure of hPrdx6 at pH 7.0 and 4.0, the (**A**) Far-UV CD, (**B**) Near-UV CD, (**C**) Tryptophan fluorescence and (**D**) ANS binding assay measurements are done.

Impact of change in solution pH on hPrdx6’s conformation is probed by using various spectroscopic tools including circular dichroism (CD) and fluorescence spectroscopy (See Fig. 2). Far-UV CD (Fig. 2A and Table 1) reveals that there is reduction in the secondary structural content of hPrdx6 at pH 4.0 (as compared to pH 7.0) specifically in the θ_222_ region which is an indication of the alteration in the alpha helix. Similarly, Near-UV CD shown in Fig. 2B also suggest a gross change in the tertiary structure because of the observed decrease in CD signals at θ_269,_ θ_279,_ and θ_289_ regions. It is also seen in Fig. 2C that tryptophan fluorescence spectra has been red shifted followed by large reduction in fluorescence intensity at λ_max_ indicating certain chromophore(s) that was buried in the core of the protein have been shifted to the polar environment. We have further investigated if the altered tryptophan micro-environment has affected the exposition of buried hydrophobic groups. ANS binding assay shown in Fig. 2D infer that although there is slight ANS binding with a blue shift observed in hPrdx6 at pH 7.0, the ANS binding propensity of hPrdx6 radically increases at pH 4.0. The results indicate that many of the hydrophobic groups have been solvent exposed under acidic condition.

**Table 1:** Secondary structure content of hPrdx6 at pH 7.0 and 4.0.

	pH 7.0 (%)	pH 4.0 (%)
Alpha helix	29.2	22.8
Beta sheet	27.6	26.4
Others	43.2	50.8

### Switching from pH 7.0 to acidic condition induce oligomerization of hPrdx6

**Figure 3:**
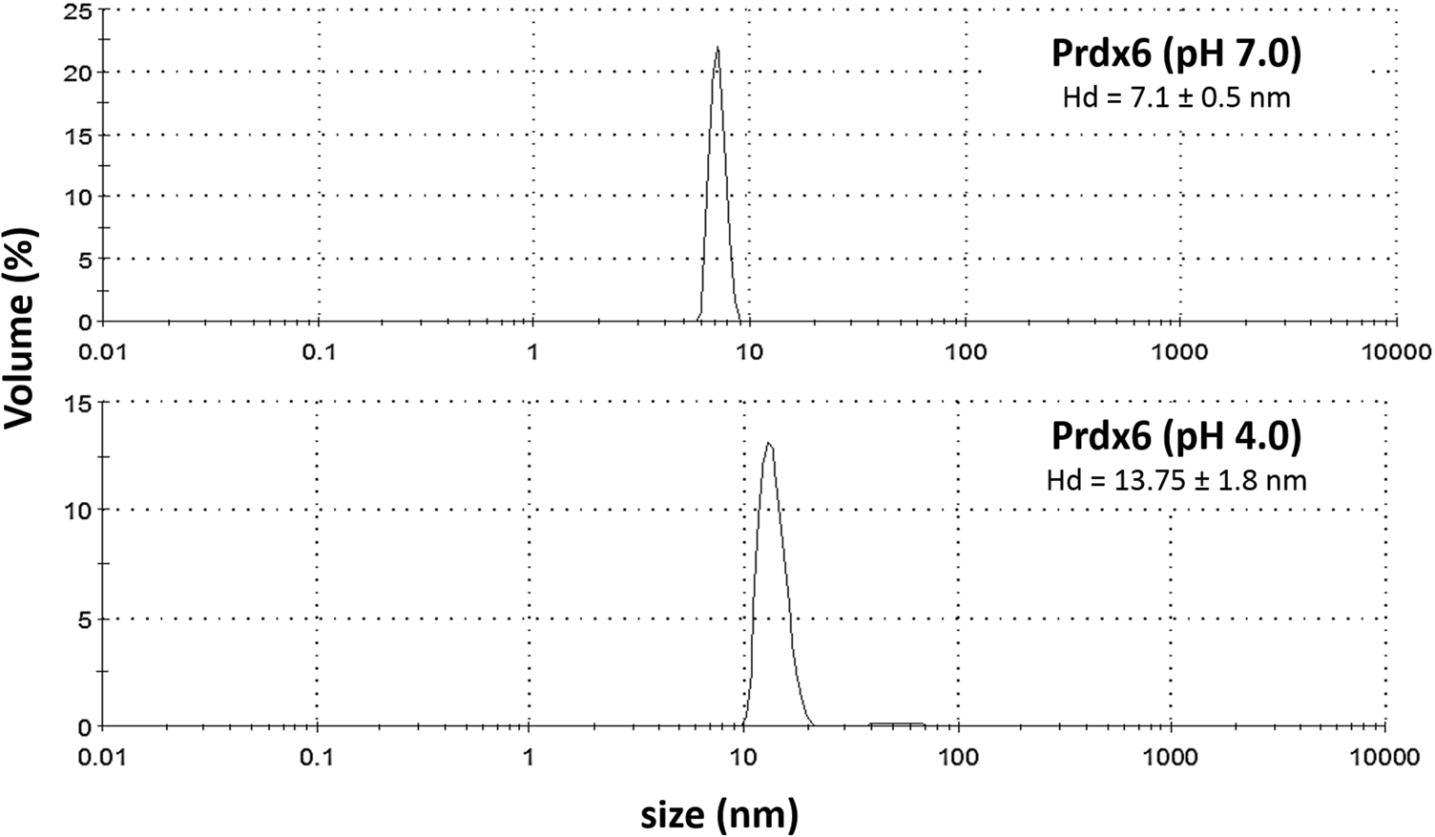
Dynamic light scattering (DLS) measurement of hPrdx6. The size distribution by volume of hPrdx6 at pH 7.0 and 4.0 is determined using DLS. The size measured here is reflective of the hydrodynamic diameter (in nm) of the protein.

**Figure 4:**
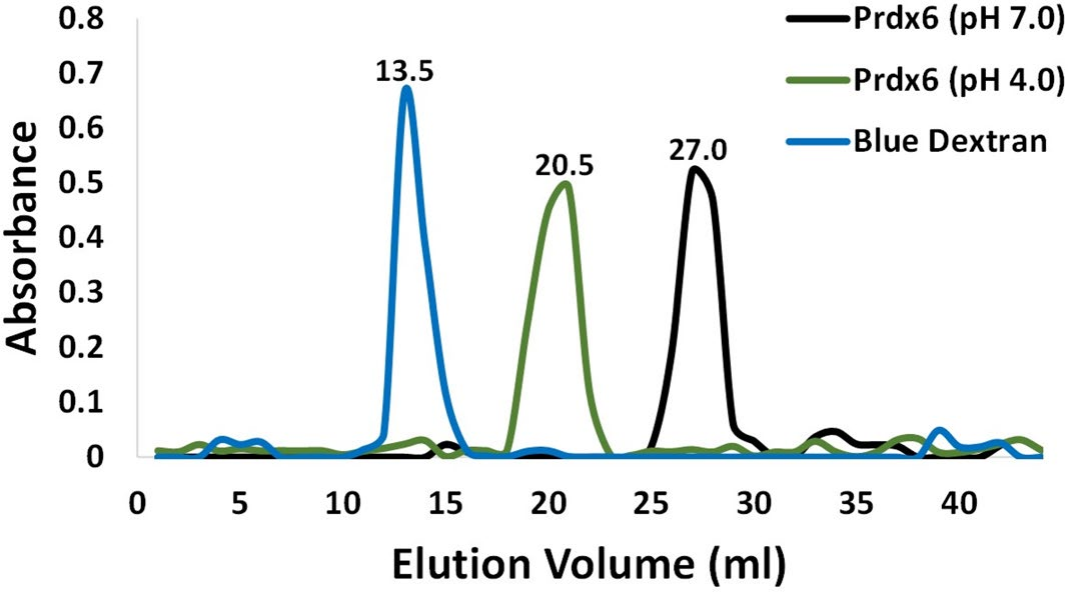
SEC profiles of Human Prdx6 at cytosolic and lysosomal pH. The hPrdx6 (monitored via absorbance measurement at 280 nm) during size exclusion chromatographic analysis was found to elute at 27.0 ml and 20.5 ml at pH 7.0 and pH 4.0, respectively.

The oligomeric status of the protein at pH 7.0 and 4.0 was investigated with the help of DLS. The plot of hydrodynamic diameter (H_d_) of hPrdx6 at pH 7.0 and 4.0 versus percentage volume occupancy in the solution as measured via DLS is shown in Fig. 3. It is seen in this figure that at pH 7.0, hPrdx6 displayed H_d_ of ~ 7 nm, corresponding to that of a dimer with a molecular mass approximately 50 kDa. The H_d_ is found to be almost doubled at pH 4.0, roughly corresponding to that of a protein with100kDa, indicating that the switching of protein microenvironment from cytosolic to lysosomal pH results in dimer to tetramer transition in hPrdx6’s oligomeric status. To reaffirm our findings, we also performed SEC with hPRDX6 at both lysosomal and cytosolic pH. Figure 4 shows elution profiles (absorbance versus elution volume) of Blue Dextran, and hPrdx6 at different pH on sephadex G-100 column. As seen in the figure, all these components eluted from the column in the form of a single symmetrical peak. Elution volume of the blue coloured fractions of blue dextran monitored at 600 nm represented void volume (13.5 ml) of the column. The protein, hPrdx6 at pH 7.0 and 4.0, when monitored at 280 nm, eluted after the blue dextran peak with an elution volume of 27 ml and 20.5 ml, respectively. This clearly shows that molecular weight of hPrdx6 at acidic pH is more than hPrdx6 at cytosolic pH. This observation is consistent with our data from DLS study which shows hPrdx6 to be a dimer of molecular weight 50 kDa and a tetramer of approximately 100 kDa at pH 7.0 and 4.0, respectively.

### Correlation between functional activity and nature of oligomers

**Figure 5:**
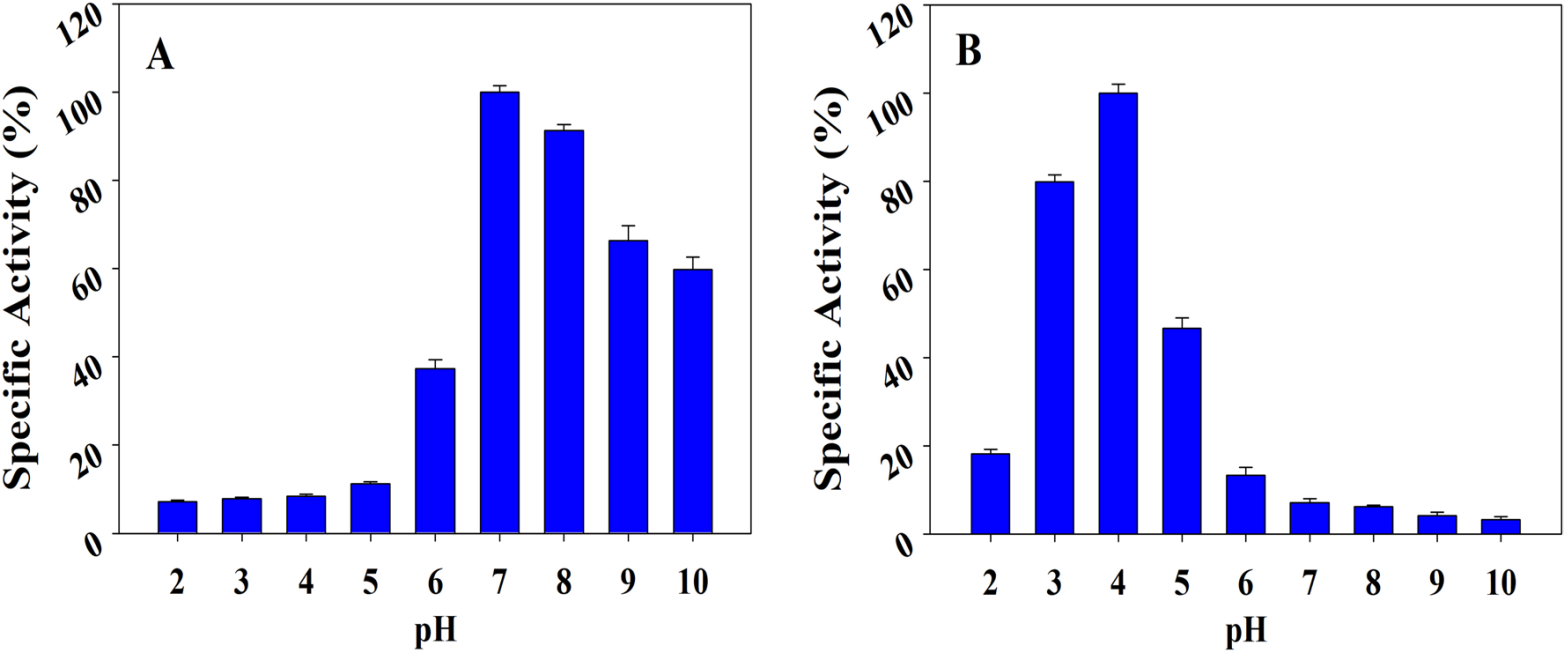
Effect of pH (2.0–10.0) on hPrdx6’s glutathione peroxidase and aiPLA_2_ activity. (**A**) Plot of specific glutathione peroxidase activity (%) versus pH. The percent specific activity of the enzyme at pH 7.0 is considered as 100%, and (**B**) Plot of specific calcium independent phospholipase A2 activity (%) versus pH. The percent specific activity of the enzyme at pH 4.0 is considered as 100%.

We have first of all performed measurement of oligomeric nature of hPrdx6 at a range of pH values (pH 2.0–10.0) (see Supplementary Fig. S2). It is seen in Table 2 that starting from pH 10.0 to 6.0, there is no significant difference in the H_d_ of the protein indicating that the dimeric state is maintained. However, at pH 5.0, the protein exists in dimer-tetramer equilibrium (30% dimer and 70% tetramer) beyond which it purely exists in its tetrameric state till pH 3.0. At pH 2.0 the protein now apparently exists in monomer–dimer equilibrium (70% monomer and 30% dimer). Thus different pH conditions help to determine the oligomeric state of the protein. To compare these oligomeric properties with the functional activity of the protein, we also carried out systematic measurement of peroxidase and aiPLA2 activity of hPrdx6 at the similar pH conditions where the oligomers were analyzed (See Fig. 5). It is seen in Table 2 that as expected upto pH 3.0 to 5.0 (where the protein exists in tetrameric state), there is no peroxidase activity but predominantly shows aiPLA2 activity. However, at pH 6.0 (where the protein is in the dimeric state) and above, the activity is completely dominated by the peroxidase function. At pH 2, the enzyme barely exhibits some aiPLA2 activity but devoid of peroxidise activity. Thus, there is a good correlation between the pH-induced oligomeric state and the nature of functional state.

**Table 2:** Effect of different pH (2.0–10.0) on hPrdx6’s hydrodynamic diameter (Hd).

	Hd (nm)	Volume fraction (%)	Polydispersity index
pH 10.0	6.3 ± 0.3	100	1.0
pH 9.0	6.4 ± 0.9	100	0.372
pH 8.0	6.31 ± 0.8	100	0.437
pH 7.0	7.1 ± 0.5	100	0.437
pH 6.0	8.12 ± 0.6	100	0.276
pH 5.0	6.39 ± 0.7	29.8	0.353
	13.44 ± 0.5	70.2	
pH 4.0	13.75 ± 1.8	99.6	0.275
pH 3.0	12.2 ± 3.0	99.2	0.712
pH 2.0	14.17 ± 1.7	30.2	0.395
	3.7 ± 0.7	69.8

## Discussion

Development of moonlighting proteins is believed to be the result of evolution through which uni-functional proteins gained the ability to perform multiple functions to conserve amino acids and energy required to synthesize multiple proteins^17^. Interestingly, the differential functional behaviour of many of these proteins depends not only on the structural allostery, but also their cellular locations. hPrdx6 is a prime example of the moonlighting protein exhibiting location specific multi-functionality. In general, most probable triggers for this kind of location dependent functional moonlighting are (1) differential post-translational modifications, (2) variable concentration of protein’s ligand/substrate in different locations, or (3) conformational alteration brought about by the altered micro-environment. Since moonlighting behaviour of hPrdx6 is triggered by the micro-environment (i.e., cytosolic and lysosomal), the primary difference is pH (pH 7.0 for cytosol and 4.0 for lysosome). Therefore, our main objective is to unveil if altered pH brings changes in the structure, stability or oligomeric nature of hPrdx6 to induce functional switch. Our results on the measurement of thermodynamic stability of the protein indicate that hPrdx6 stability is different at the two different pH values (Fig. 1). It is evident in Fig. 1A that the protein is more stable at low pH than that at pH 7.0. However, upon heat-induced denaturation (Fig. 1B), the protein could not be unfolded within the measurable temperature range indicating that sensitivity of the protein against the two modes of denaturants is different. Interestingly, enhanced protein stability at low pH as a strategy to avoid acid induced unfolding has also been observed in many proteins including cytokines, interleukin-2 (IL-2) and granulocyte colony stimulating factor (G-CSF), which usually get internalised in acidic endosomal compartments, and capsid proteins which ensure virus’s survival in acidic intestinal tract^18–20^. Since hPrdx6 exhibits aiPLA_2_ activity at low pH, the conformational resistance against very high temperatures might implicate the evolutionary importance of aiPLA_2_ in thermophiles. In support of this argument, hPrdx6 has been characterized in certain extremophiles^21–24^. However, the advantages of its aiPLA2 or peroxidase activity in these organisms have not been explicitly explored yet.

**Figure 6:**
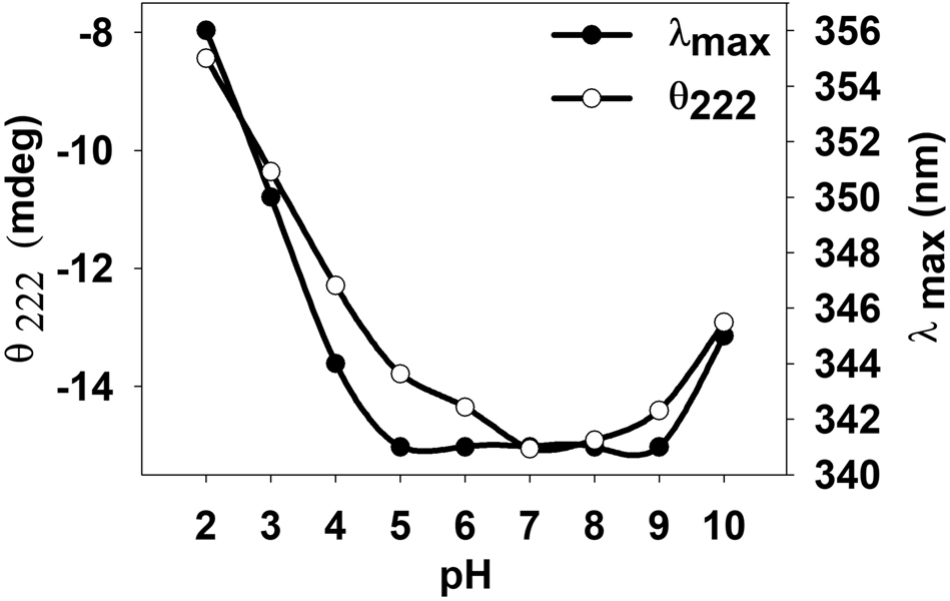
Effect of pH (2.0–10.0) on secondary and tertiary structure of hPrdx6. The effect of change in pH from 2.0 to 10.0 on the secondary and tertiary structure of hPrdx6 determined using Far-UV CD, and Near-UV CD is indicated by plotting the change in ϴ_222_ and λ_max_ with escalation of pH 2.0 to 10.0 by the unit of 1.

Next, we investigated the structural differences (at pH 7.0 and 4.0) that is making variation in the stability of hPrdx6 using multiple spectroscopic probes (Fig. 2). Far UV CD measurement (Fig. 2A) indicates that at low pH, there is decrease in the secondary structural elements exemplified by the large decrease in alpha helix and a small change in beta with corresponding increase in random coil fractions (Table 1). These changes in the secondary structure brings about large alterations in the tertiary structure of the protein at low pH as evidenced from the gross decrease in the near UV CD and micro-environment of tryptophan (due to hypochromicity with shift in λ_max_). An alteration in structure with decrease in secondary and tertiary elements might result in low packing efficiency in the native state leading to exposition of some of the hydrophobic groups to the solvent. We use ANS binding assay to explore this possibility as ANS is a hydrophobic dye that specifically binds to the exposed hydrophobic clusters in proteins. As evident in Fig. 2D, there is large binding of ANS at low pH but very minimal at physiological pH indicating that many hydrophobic groups have been exposed to the solvent. Taken together, the results indicate that low pH induces conformationally unstable native state as compared to pH 7.0. We have further monitored the structural details at a range of pH’s (pH 2.0–10.0) to further unfold the pH-induced structural alterations by measuring far UV CD and tryptophan fluorescence (Supplementary Fig. S3). A plot of θ_222_ versus pH and λ_max_ versus pH is shown in Fig. 6. It is seen in this figure that both probes yield nearly identical pattern of conformational transitions. With gradual decrease in pH (from physiological condition), there is corresponding loss of secondary and tertiary structures. Similarly, with increase in pH toward alkaline conditions, the protein experiences similar pattern of structural loss. The results further substantiate that changes in the conformation are gradual with pH titrations.

Indeed, the conformational alterations observed here do not correlate with the results on thermodynamic stability. Our speculation is that enhanced thermodynamic stability despite reduction in secondary and tertiary structure (at pH 4.0) might be the result of higher order oligomer formation (at acidic pH) as compared to the physiological condition because exposition of hydrophobic cluster might help to initiate an oligomerization step. For instance, acidic pH has been reported to trigger oligomerisation of pulmonary surfactant protein B by inducing formation of a protein folding motif stabilised by hydrophobic interactions^25^. Analogously, Prdx1, also shows a low pH induced oligomeric switch such that at acidic pH of 4.4 it favours decameric form and a dimer at high pH^26^. In order to substantiate this claim, we performed DLS and SEC measurement to analyse oligomeric nature of the protein samples at pH 4.0 and 7.0 (Figs. 3 and 4). The hPrdx6 has been reported to be a dimer in existence at physiological pH^27^. Our DLS and SEC studies therefore, indicate that the dimer has been converted to a tetrameric species as the hydrodynamic diameter is roughly increased to twice its size. We have further ascertained the respective activities of the dimer and tetramer by performing peroxidase and aiPLA2 activity assay (Fig. 5) respectively. Thus, we conclude that the acidic pH induces unique secondary and tertiary structure that is competent to form higher order oligomer. In agreement, analysis of crystal structure of hPrdx6 revealed that, hPrdx6 forms dimer because of important hydrophobic contacts mediated by Phe43, Phe45, Trp82, Tyr89, Leu145, Ile147, Leu148, Tyr149, Val179, Pro191, Tyr217, and Tyr220 residues^27^.

Results from conformational measurements also suggest that dimeric hPrdx6 is conformationally very unstable in lysosome or lamellar bodies. High order oligomer at low pH condition (in lysosome), therefore, perhaps provides an advantage to the enzyme to stay protected against acid-induced destabilization/inactivation and enhance longevity in the protein degradation organelle. In agreement to this, there are various organisms like Herpes simplex virus, prions, *Acidithiobacillus Ferrooxidans*, *Sulfolobus Solfactaricus* in whom acid-resistance property of their proteins is correlated with enhanced hydrophobicity and oligomerization^23,28,29^. Reduced solvent accessibility of these proteins due to oligomerisation at acidic pH is believed to contribute towards entropic stabilization by increasing amino acid contact density^28,30,31^.

We were further interested to examine how the quaternary structure changes with respect to the different pH ranges (pH 2–10) correlate with the change in activity status. For this we made systematic activity and DLS measurements at different pH ranges. As evident in Fig. 5 and Table 2, there is a strong correlation between the pH, quaternary structure and two enzymatic activities of hPrdx6. In the pH range (pH 6.0–10.0) hPrdx6 exists as dimer and possess glutathione peroxidase activity, while the appearance of tetrameric species in the acidic pH conditions (pH 3–5) coincided with the disappearance of peroxidase and appearance of aiPLA_2_ activity. The loss of both activities at pH 2.0, however, seems to have arisen because of the breakdown of hPrdx6 tetramers into monomers (Fig. 5 and Supplementary Fig. S2) and subsequent unfolding^32^. Correspondingly, the alterations seen in the secondary and tertiary structure of hPrdx6 across the range of pH 2–9 (Fig. 6 and Supplementary Fig. S3) also seem to agree well with that of the quaternary structure changes observed in the DLS experiments. It is to be noted here that in case of pH 10.0, although the secondary and tertiary structure of hPrdx6 differs from that of the cytosolic conformer, it remained dimeric and functionally peroxidase active, thereby buttressing the importance of quaternary structure in determining the functional property of hPrdx6 enzyme. Thus, we confirm that pH plays a key role in regulating the structure and function of hPrdx6.

## Summary

Low pH induces alteration in secondary and tertiary structures that is sufficient to form high order oligomer. The formation of this oligomer appears to be the rationale for structural resistance of the hPrdx6 against acid-induced denaturation in lysosome or lamellar bodies. The correlation between pH, quaternary structure and two enzymatic activities of hPrdx6 also strongly indicates the change in quaternary state of the enzyme is the most probable reason for the pH specific nature of its two moonlighting functions. The hPrdx6 at acidic condition is also observed to gain thermal resistance indicating the importance of aiPLA2 activity in various extremophiles. This avenue should therefore, be addressed and expanded in future.

## Supplementary Information

Supplementary Figures.
