# Supplementary material for: pH induced conformational alteration in human peroxiredoxin 6 might be responsible for its resistance against lysosomal pH or high temperature
Source: Sci Rep. 2021 May 6;11:9657. doi: 10.1038/s41598-021-89093-8 (PMC8102515; doi:10.1038/s41598-021-89093-8)

**SUPPLEMENTARY INFORMATION**

**pH induced conformational alteration in human peroxiredoxin 6 might be responsible for its resistance against lysosomal pH or high temperature**

Rimpy Kaur Chowhan^1^, Sunaina Hotumalani^1^, Hamidur Rahaman^2^, LaishramRajendrakumar Singh^1,*^

*^1^ Dr. B.R. AmbedkarCenter for Biomedical Research, University of Delhi, Delhi, India 110007*

*^2^ Department of Biotechnology, Manipur University, Imphal, India 795003*

^*^**Corresponding author**: Laishram R. Singh. Tel: +91-9811630757; E-mail: [lairksingh@gmail.com](mailto:lairksingh@gmail.com).

**Running title:** pH induced conformational alteration in Prdx6

**Figure S1: SDS-PAGE analysis of purified hPrdx6.** The purified hPRDX6 when analysed using SDS-PAGE was observed to be devoid of any impurity with a single band corresponding to monomeric units of ~26kDa. (The Gel marker bands from top to bottom in the first lane corresponds to molecular weights: 250 kDa, 150 kDa, 100 kDa, 75 kDa, 50 kDa, 37 kDa, 25 kDa and 20 kDa, respectively.)

**
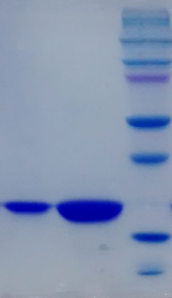
**

**Figure S2: Dynamic light scattering (DLS) measurement of Prdx6 at different pH (2.0 to 10.0).** The volume size distribution plot of Prdx6 at the pH range of 2.0 to 10.0 is determined using DLS. The size measured here is reflective of the hydrodynamic diameter (in nm) of the protein. Occurrence of two peaks at a particular pH signifies presence of two different oligomeric species of Prdx6 at that pH.


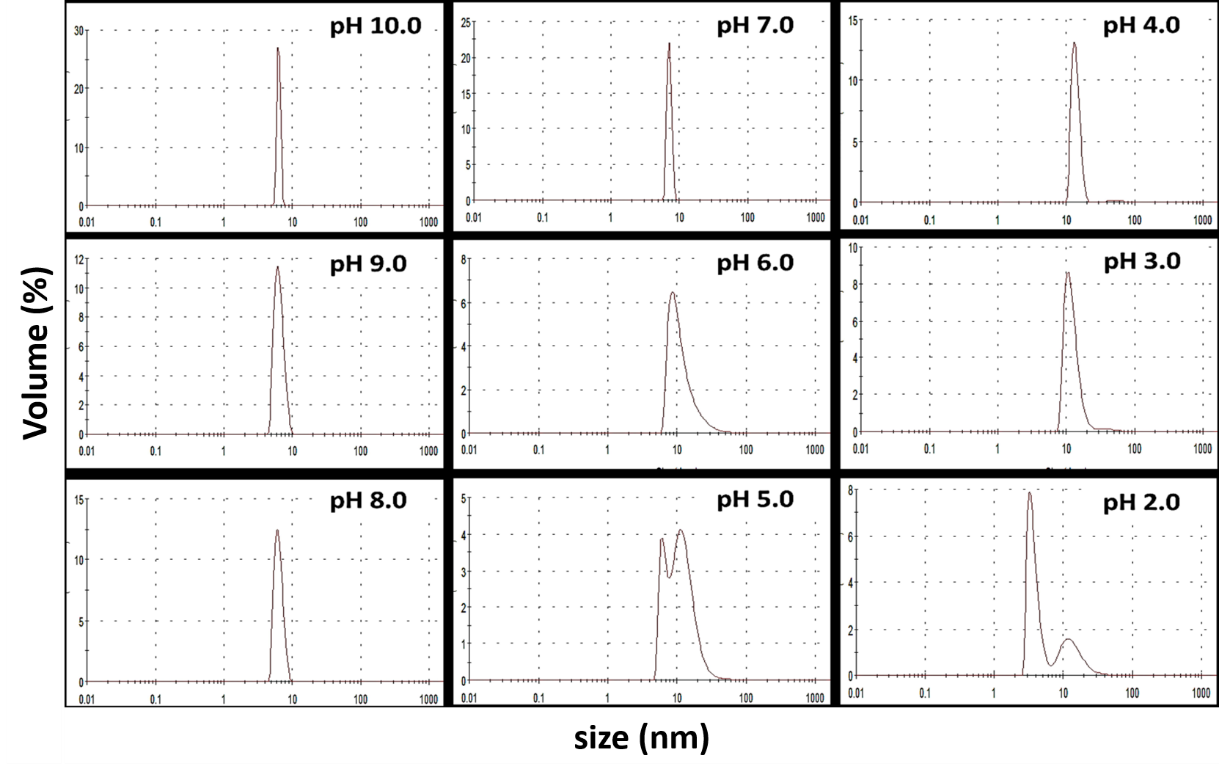


**Figure S3: Structure estimation of Prdx6 at different pH.**The secondary and tertiary structure measurement of human Prdx6 at pH 2.0-10.0 is done using (A) far-UV CD, and (B) Near-UV CD, respectively.


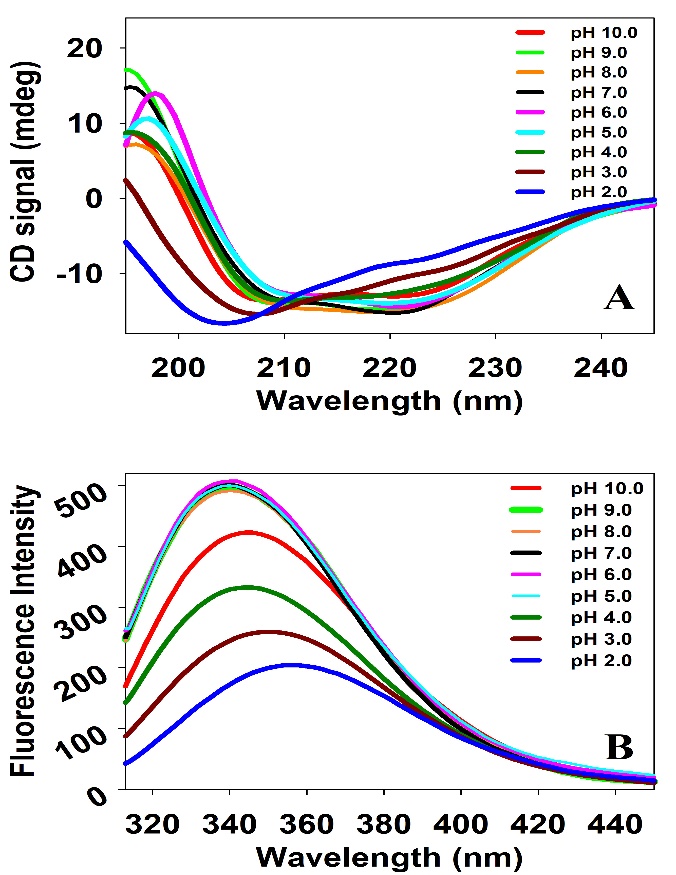

Supplement: Supplementary file 1 — Supplementary Figures. [file 41598_2021_89093_MOESM1_ESM.docx]
